# Gut microbe and hepatic macrophage polarization in non-alcoholic fatty liver disease

**DOI:** 10.3389/fmicb.2023.1285473

**Published:** 2023-12-06

**Authors:** Yao Chen, Yumeng Gan, Huijie Zhong, Yincong Liu, Jingdi Huang, Wenxue Wang, Jiawei Geng

**Affiliations:** ^1^Department of Infectious Disease and Hepatic Disease, First People’s Hospital of Yunnan Province, Affiliated Hospital of Kunming University of Science and Technology, Kunming, Yunnan, China; ^2^School of Medicine, Kunming University of Science and Technology, Kunming, Yunnan, China; ^3^School of Basic Medicine, Yunnan University of Chinese Medicine, Kunming, Yunnan, China; ^4^Faculty of Life Science and Technology, Kunming University of Science and Technology, Kunming, Yunnan, China

**Keywords:** gut microbes, translocation, hepatic macrophages, Kupffer cells, NAFLD

## Abstract

Non-alcoholic fatty liver disease (NAFLD) is a common chronic hepatic disorder with the potential to progress to hepatic fibrosis, hepatic cirrhosis, and even hepatocellular carcinoma. Activation of hepatic macrophages, important innate immune cells predominantly composed of Kupffer cells, plays a pivotal role in NAFLD initiation and progression. Recent findings have underscored the regulatory role of microbes in both local and distal immune responses, including in the liver, emphasizing their contribution to NAFLD initiation and progression. Key studies have further revealed that gut microbes can penetrate the intestinal mucosa and translocate to the liver, thereby directly influencing hepatic macrophage polarization and NAFLD progression. In this review, we discuss recent evidence regarding the translocation of intestinal microbes into the liver, as well as their impact on hepatic macrophage polarization and associated cellular and molecular signaling pathways. Additionally, we summarize the potential mechanisms by which translocated microbes may activate hepatic macrophages and accelerate NAFLD progression.

## 1 Introduction

Non-alcoholic fatty hepatic disease (NAFLD) is prevalent in 20–30% of the global population and can progress to non-alcoholic steatohepatitis (NASH, accounting for 20% of NAFLD cases), as well as hepatic fibrosis, hepatic cirrhosis, and even hepatocellular carcinoma (Dou et al., 2019). Research has also established that NAFLD can exacerbate the incidence and progression of obesity, insulin resistance, type 2 diabetes mellitus (T2DM), dyslipidemia, and cardiovascular disease (Govaere et al., 2022).

Multiple regulators influence NAFLD, including hepatic immunity (e.g., hepatic macrophages，B cells, Regulatory T cells (Treg cells)), gut microbes, lipid metabolism dysregulation, and genetic factors (Kazankov et al., 2019). Notably, activation of hepatic macrophages is closely involved in NAFLD (Slevin et al., 2020) and serves as a reliable marker of NAFLD incidence in both human patients and rodents (Kazankov et al., 2019). Strategies targeting hepatic macrophages have been shown to markedly mitigate NAFLD progression (Kazankov et al., 2019). The “two-hit” theory of NAFLD, encompassing both excessive fatty lipid accumulation and oxidative stress, is strongly associated with hepatic macrophage activation (Nagashimada and Honda, 2021).

Gut microbes are also considered crucial players in NAFLD initiation and progression. Gut microbiota dysbiosis, commonly characterized by an abnormal Firmicutes-Bacteroidetes ratio, has been reported in both animal and clinical NAFLD studies (Zhu et al., 2013). Following this dysbiosis, certain pathogenic microbes damage the intestinal barrier, increasing its permeability, and translocate to the liver via the portal vein. These translocated pathogenic microbes and their metabolites rapidly induce hepatic inflammation and macrophage polarization, consequently accelerating NAFLD progression (Mouries et al., 2019). This review outlines the pathways by which gut microbes translocate to the liver, establish colonization, and stimulate resident macrophages, leading to enhanced NAFLD progression. The goal of this review is to present novel strategies for NAFLD diagnosis and treatment.

## 2 Gut microbiota dysbiosis during NAFLD

Gut microbes significantly affect NAFLD initiation and progression. Not only do they directly metabolize substances crucial to NAFLD pathology, such as bile acids, trimethylamine, ethanol, and indole, but gut microbial dysbiosis also compromises the intestinal mucosal barrier, allowing pathogenic microbes and their metabolites to enter the portal vein from peripheral circulation, thus initiating and exacerbating NAFLD (Albillos et al., 2020). Metagenomic sequencing can provide detailed insights into the properties of the intestinal microbiota during NAFLD progression. Populations of *Escherichia coli*, *Desulfovibrio*, and *Clostridium* are markedly increased in both NAFLD patients and animal models with NAFLD-related fibrosis, while probiotic species, such as *Eubacterium rectale*, *Faecalibacterium prausnitzii*, *Akkermansia muciniphila*, *Lactobacillus*, and *Bifidobacteria*, are significantly decreased (Zhang X. et al., 2021).

Dietary structure is a significant factor in NAFLD development. For example, long-term high-fat diet (HFD) consumption disturbs intestinal microbiota, impairs the intestinal barrier, and inhibits beneficial metabolites such as short-chain fatty acids (SCFAs) and 3-indole propionic acid (IPA) (Zhang X. et al., 2021; Chassaing et al., 2022). Normal fecal rectifies intestinal microbiota disorders and enhances gut microbiota homeostasis, thereby bolsters intestinal barrier function and alleviates hepatic lipid accumulation and inflammation activities (Zhou et al., 2017). Intestinal barrier, and gut-vascular barrier (GVB), is crucial shield preventing intestinal microbiota translocation into liver (Mouries et al., 2019). Intestinal microbiota dysbiosis may lead to GVB integrity compromise. For example, NAFLD mice show elevated expression of plasmalemma vesicle-associated protein 1 (PV1), a GVB impairment marker (Mouries et al., 2019).

**Figure 1:**
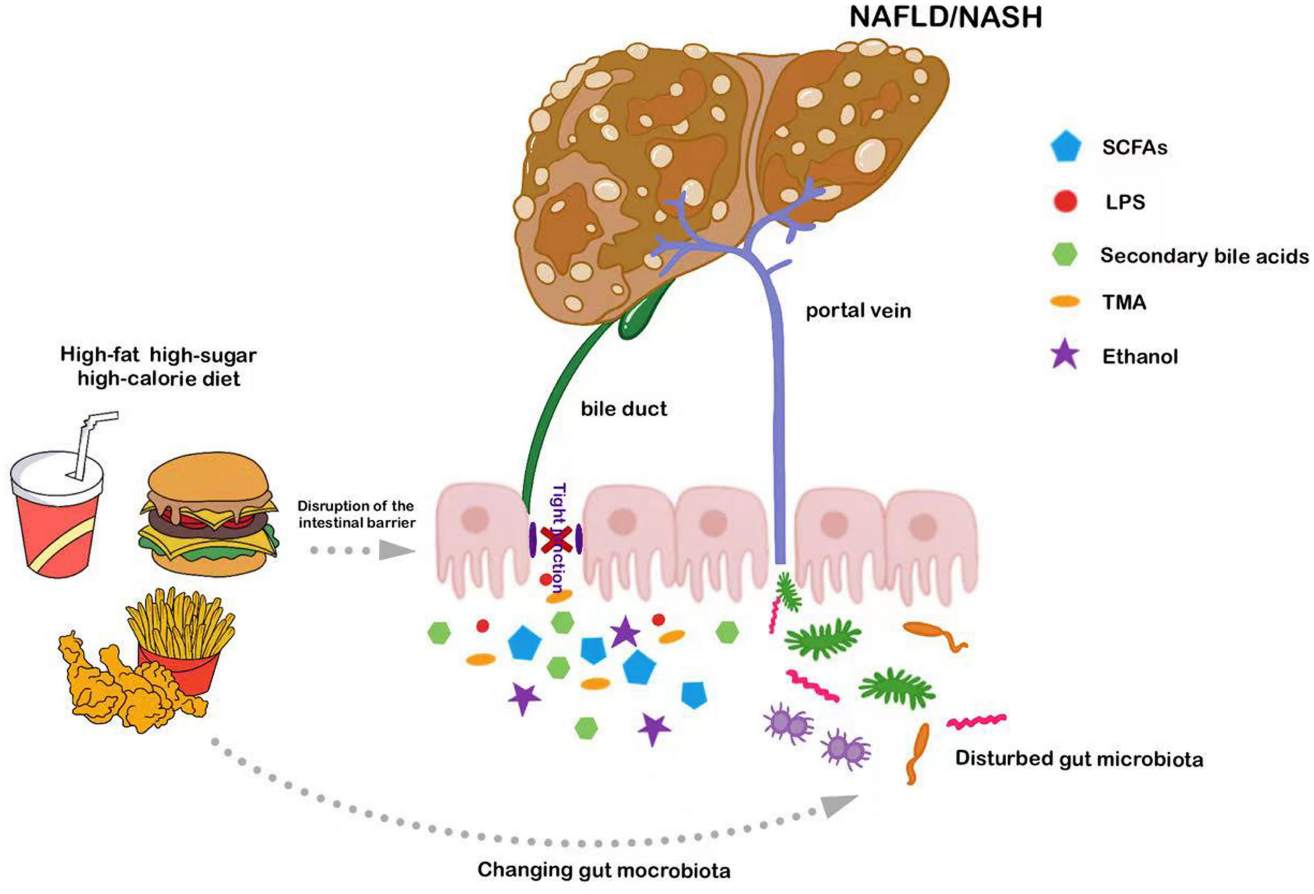
Long-term consumption of diets high in fats or sugars exacerbates NAFLD by compromising intestinal barrier function and disturbing intestinal microbiota; these disrupted microbes and their metabolites subsequently access the liver via the portal vein.

Thus, it is evident that the intestinal microbiota plays a major role in regulating hepatic metabolism and pathophysiology. Chronic consumption of a HFD induces intestinal microbiota dysbiosis and disrupts intestinal barrier function. This can, in turn, further damage the GVB and increase intestinal permeability, leading to the translocation of pathogen-associated molecular patterns (PAMPs) through the portal vein to the liver, triggering inflammation and accelerating NAFLD progression (Figure 1). Consequently, interventions aimed at modulating the intestinal microbiota present potential therapeutic avenues for NAFLD management.

## 3 Hepatic macrophage activation is a key step of NAFLD initiation and progression

Liver diseases, such as NAFLD, are tightly related to resident immunity, that composed of macrophages, B cells, and Regulatory T (Treg) cells (Barrow et al., 2021; Zhang S. et al., 2021). Hepatic macrophages, including Kupffer cells (KCs), hepatic-resident macrophages, and monocyte-derived macrophages (MoMφs), serve as primary defense mechanisms against bacterial incursions (Wen et al., 2021). Activated KCs secrete chemokines to recruit inflammatory MoMφs into liver and activate rest immune cells, consequently exacerbating local inflammatory response and liver injury (Kazankov et al., 2019). Depending on the state of the microenvironment, hepatic macrophages can differentiate into two distinct types, i.e., classically activated M1 pro-inflammatory type and alternatively activated M2 anti-inflammatory type. Under normal conditions, macrophages, influenced by Th2 cytokines IL-4 and IL-13, adopt the M2 phenotype, utilizing IL-10, arginase 1 (Arg1), transform growth factor-β (TGF-β), and other anti-inflammatory factors to reduce inflammation and facilitate tissue repair. In contrast, macrophages exhibiting the M1 but frequently intensify inflammatory diseases by releasing TNF-α, IL-1β, and CCL2, contributing to systemic insulin resistance and advancing hepatic disease (Wang C. et al., 2021). The polarization between M1 and M2 macrophages is dynamic. During the repair stages of NAFLD and NASH, hepatic M1 macrophages can be replaced by M2 macrophages, which exhibit immunosuppressive and profibrotic effects (Wang C. et al., 2021). Therefore, strategies that focus on modulating the activation and phenotypic shifts of hepatic macrophages offer promising therapeutic approaches for NAFLD management.

Hepatic macrophages can be activated by various agents, such as ethanol, fatty acids, and oxidative stress, with lipopolysaccharide (LPS) serving as a notable exogenous activation stimulus (Krenkel et al., 2018; Nagashimada and Honda, 2021). Intestinal permeability is a key determinant of NAFLD development, gut-derived LPS through the portal vein arrive in the liver drain bind to its natural ligand TLR4 and activates the downstream nuclear factor kappa-B (NF-κB) through the myeloid differentiation factor 88 (MyD88)-dependent signaling pathway, inducing the expression of TNF-α, IL-1, IL-6 and other inflammatory cytokines, and promoting M1 macrophages activation (Kazankov et al., 2019). In addition, LPS further interacts with Notch1 via MyD88-dependent or independent pathways, promoting the secretion of pro-inflammatory factors, such as IL-6 and inducible nitric oxide synthase (iNOS), and fostering hepatic M1 macrophage polarization (Chen et al., 2021). Thus, LPS is a key factor in directing hepatic macrophages toward the M1 phenotype. Lipid accumulation in hepatocytes is a typical histological feature of NAFLD, representing the “first strike” in NAFLD pathophysiology (Wu et al., 2020). Saturated fatty acids engage the classical TLR4/NF-κB signaling pathway to activate M1 macrophages (Wu et al., 2020). They also disrupt other cellular signaling pathways, leading to the release of mitochondrial DNA (mtDNA) into the cytoplasm, which binds to NOD-like receptor thermal protein domain associated protein 3 (NLRP3) to form inflammatory vesicle complexes. Activation of NLRP3 inflammasomes in KCs promotes the secretion of IL-1β, exacerbates the inflammatory response, and enhances hepatocyte injury (Pan et al., 2018). Oxidative stress is another well-recognized factor in NAFLD development, representing the “second strike” in NAFLD pathophysiology. It not only stimulates M1 macrophage polarization, but also increases the sensitivity of macrophages to LPS (Nagashimada and Honda, 2021). These observations reinforce the potential of antioxidant therapy as a direct measure to counteract oxidative stress and guide the transition of hepatic macrophages from the M1 to M2 phenotype. For example, in NAFLD mice, the natural antioxidant resveratrol has been shown to modulate the TLR4/NF-κB signaling pathway, attenuating activation of hepatic M1 macrophages and reducing expression of pro-inflammatory factors 18. In NASH animal models, the flavonoid myricetin has been observed to inhibit interactions between the TREM-1-TLR2/4-MyD88 signaling pathway and hepatic M1 macrophages, leading to decreased hepatic inflammation and fibrosis (Yao et al., 2020).

B lymphocyte, an integral component of the adaptive immune system in liver, is also a pivotal factor contributing to NASH progression (Bruzzì et al., 2018). In NASH, pro-inflammatory B2 cell accumulation in liver has been demonstrated to result in increased expression of pro-inflammatory cytokines, such as TNF-α and IL-6 through TLR4/MyD88 signaling pathway, that ultimately exacerbating liver inflammation and fibrosis. Moreover, depletions of B2 cells or B cell-specific MyD88 significantly ameliorate the NASH progression (Barrow et al., 2021). Additionally, Treg cell is also considered to play an indispensable role in the NAFLD progression (Zhang S. et al., 2021). In animal and clinical studies of NAFLD, a decreased number of Treg cells have been observed. The increase of T helper 17 (Th17) to Treg cell ratio in peripheral blood and liver tissue is a clinically indicative of NAFL progression toward NASH (Riaz et al., 2022). Adoptive transfer of Treg cells leads to a reduction in TNF-a expression and mitigates hepatic inflammation (Zhang S. et al., 2021). However, excessive immunosuppression impairs normal immune function. For instance, Accumulated Treg cells, have been observed in Hepatocellular carcinoma (HCC). Both AKT/Ras and c-Myc interact with the CCL2-NF-kB axis in macrophages, and thereby promote e immunosuppression and immune escape of tumor cells (Liu et al., 2022).

Even both B cells and Treg cells are involved in the NAFLD progression, their specific functions are inextricably linked to macrophages. CyTOF data revealed a pronounced enrichment of monocytes and macrophages in the high-fat/high-cholesterol (HFHC)-fed NAFLD mice livers, followed by the increased infiltration of B cells, natural killer (NK) cells, and dendritic cells (DCs) (Barrow et al., 2021). In another hand, inflammation signaling plays a pivotal role in the NASH progression. The Myd88-TLR4 signaling pathway serves as a crucial factor in activating B cells to promote NASH. LPS, a TLR-related agonist, stimulates macrophages, B cells, and neutrophils, and finally promotes inflammatory cytokine secretions, such as TNF-α and IL-6 by macrophages (Barrow et al., 2021). Additionally, Treg cells is partially reliant on MHC-II, because liver macrophages and B cells serve as crucial subsets of liver antigen-presenting cells (APCs) (Riaz et al., 2022).

**Figure 2:**
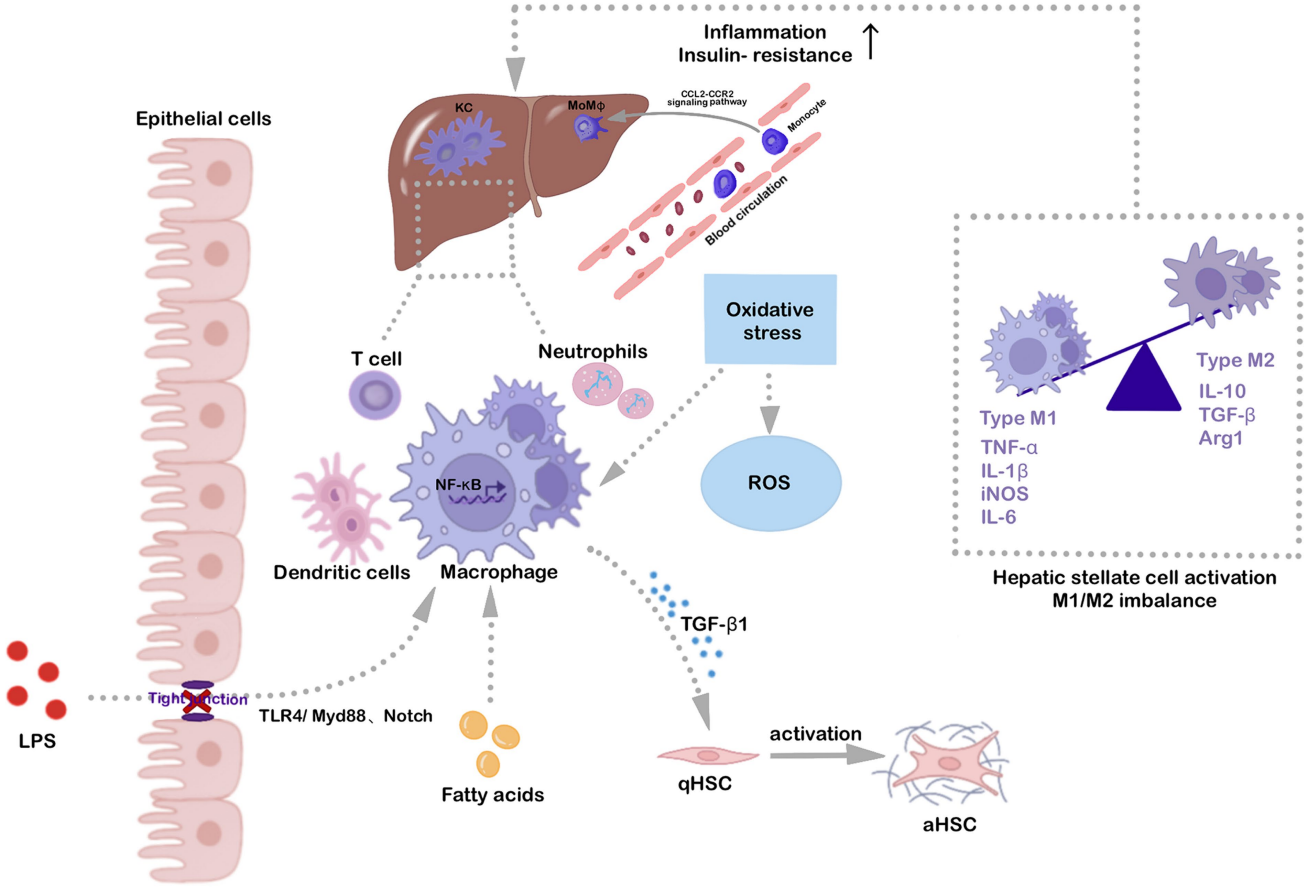
Hepatic macrophage activation is a key factor in NAFLD/NASH progression. Fatty acids, oxidative stress, and gut-derived LPS activate hepatic-resident KCs through the classical TLR4/MyD88/NF-κB signaling pathway. The Notch signaling pathway also contributes to macrophage polarization. Specifically, LPS up-regulates Notch1 expression via activation of macrophage MyD88-dependent or independent pathways, fostering pro-inflammatory cytokine release and hepatic macrophage activation. Activated KCs also induce circulating monocytes to the liver via the CCL2-CCR2 signaling pathway, converting them to inflammatory MoMφs. Various inflammatory stimuli are involved in hepatic macrophage activation, promoting polarization toward the M1 phenotype and secretion of pro-inflammatory cytokines, including TNF-α, IL-1β, IL-6, and iNOS, which aggravate inflammation and insulin resistance. Activated macrophages also induce hepatic stellate cell activation, which is associated with liver fibrosis development, ultimately aggravating NAFLD/NASH.

The activation of liver macrophages is the early and pivotal event in NAFLD pathogenesis, which simultaneously affects the activation and functionality of other immune cells in the liver. The liver contains two major types of macrophages, i.e., KCs and MoMφs (Wen et al., 2021). In response to liver damage signals, KCs are activated by LPS, fatty acids, and oxidative stress and interact with the classical TLR4/MyD88/NF-κB signaling pathway to differentiate toward the M1 phenotype, resulting in increased secretion of TNF-α, IL-6, IL-8, and iNOS, which promote inflammation and NAFLD progression. Simultaneously, circulating monocytes can transform into pro-inflammatory MoMφs, influencing the balance between M1 and M2 phenotypes through the CCL2-CCR2 signaling pathway (Nagashimada and Honda, 2021). Therefore, targeting the key elements and signaling pathways involved in hepatic macrophage activation to modulate their phenotype may be a promising avenue to alleviate inflammation and halt the progression of NAFLD/NASH to fibrosis (Figure 2).

## 4 Gut microbes directly and indirectly regulate hepatic microphage polarization

Following intestinal leakage and microbial dysbiosis, intestinal microbes and their metabolites are transported to the liver through the gut-liver axis where they interact with pattern recognition receptors (PRRs) on the surface of hepatic macrophages, activating cascade inflammatory signaling pathways, promoting M1 macrophage polarization, and aggravating hepatic injury in both patients and animal models with NAFLD (Aron-Wisnewsky et al., 2020). This mechanism underscores the critical role of intestinal microbes and their metabolites in the development and progression of liver disease through the regulation of hepatic macrophage polarization.

### 4.1 *Escherichia coli* induces hepatic macrophage polarization to M1 phenotype

*Escherichia coli* is a significant gram-negative opportunistic pathogen in the human body. Emerging evidence has demonstrated that *E. coli*, enriched in the intestines of NAFLD patients, is crucial for NAFLD progression (Zhang et al., 2020). Notably, *E. coli*-related LPS drains through the portal vein and arrives in the liver through the TLR4/MyD88/NF-κB signaling pathway, driving the transition of hepatic macrophages from the M1 to M2 state in patients and animal models with NAFLD and ultimately aggravating liver injury (Carpino et al., 2020).

In NASH patients, an increase in the *E. coli* strain NF73-1 in feces and intestinal mucosa has been linked to liver pathology. Studies, both *in vivo* and *in vitro*, have demonstrated the translocation of *E. coli* NF73-1 to the liver, with subsequent activation of hepatic M1 macrophages via the TLR2-NF-κB/NLRP3-caspase-1 signaling pathway. These activated M1 macrophages, through the mTOR-S6K1-SREBP-1 signaling pathway, amplify disturbances in hepatic lipid metabolism in NAFLD mice, exacerbating liver injury and promoting NAFLD progression (Zhang et al., 2020). In the context of cirrhosis, an advanced stage of NAFLD, outer-membrane vesicles from *E. coli* have been shown to induce C-type lectin domain family 4 member E (Clec4e) expression in hepatic macrophages and neutrophils, which influences hepatic immunity, activates hepatic M1 macrophages, and exacerbates cirrhosis development (Natsui et al., 2023).

### 4.2 Probiotic, *Akkermansia muciniphila*, inhibits hepatic macrophage polarization to M1 phenotype

*Akkermansia muciniphila*, the sole representative species of the Verrucomicrobia phylum found in the human intestine and an emerging next-generation probiotic, constitutes approximately 1–3% of the healthy human intestinal microbiota (Cani et al., 2022).

NAFLD manifests from disruption of the gut-liver axis, compromised intestinal barrier, altered intestinal microbiota, hepatic lipid accumulation, and immunological imbalance. The relationship between T2DM and NAFLD is highlighted by a notable decrease in *A. muciniphila* abundance, as evidenced in mice with NAFLD-related cirrhosis (Zhang X. et al., 2021). Studies have shown that *A. muciniphila* can strengthen intestinal epithelial development, enhance intestinal barrier, and regulate intestinal microbiota, SCFA secretion, bile acid salt uptake and tryptophan metabolism and intestinal immunity. Furthermore, these bacteria are involved in cholesterol transport and fatty acid metabolism and can down-regulate the expression of sterol regulatory element binding protein-1c (SREBP-1c), thereby ameliorating NAFLD (Han et al., 2022). Randomized controlled trials have further demonstrated that daily oral supplementation of 10^10^ *A. muciniphila* can improve insulin sensitivity in overweight and obese patients with insulin resistance, reduce circulating metabolic levels, and effectively strengthen the intestinal barrier, ultimately inhibiting inflammation and improving hepatic function (Depommier et al., 2019).

The classical TLR4/NF-κB signaling pathway activates hepatic responses and exacerbates inflammation. This pathway, along with M1 macrophage activation, can be inhibited by *A. muciniphila* administration in mice with CCL4 or HFD-induced NAFLD (Keshavarz Azizi Raftar et al., 2021). TNF-α is a critical inflammatory cytokine associated with various chronic liver diseases, including NAFLD (Potoupni et al., 2021). Produced by activated KCs and modulated by intestinal microbes, TNF-α abundance is diminished in patients with elevated Bifidobacterium levels, and intestinal microbiota can robustly suppress its expression via tryptophan metabolism (Schirmer et al., 2016). Previous studies have also revealed that pasteurized *A. muciniphila* and its extracellular vesicles (EVs) can significantly reduce hepatic TNF-α expression and serum TNF-α and IL-6 levels to resist HFD-induced hepatic injury (Keshavarz Azizi Raftar et al., 2021). Liraglutide, a glucagon-like peptide-1R (GLP-1R) agonist used in T2DM treatment, has been shown to mitigate hepatic inflammation by inhibiting circulating macrophage markers and TNF-α, while *A. muciniphila* secretes glucagon-like peptide-1 (GLP-1) to participate in blood glucose regulation (Somm et al., 2021).

### 4.3 Another famous probiotic, *Lactobacillus* bacteria, contribute to hepatic macrophage polarization to M2 phenotype

*Lactobacillus* plays a crucial role in the intestinal microbiota as primary probiotics, ameliorating NAFLD via modulation of the intestinal microbiota and their metabolites and attenuation of oxidative stress and hepatic inflammation. Notably, probiotic mixtures containing *L. acidophilus*, *L. casei*, *L. reuteri*, and *Bacillus coagulans* have been shown to substantially reverse the serum and liver triglyceride levels and inhibit hepatic oxidative stress, which is critical for liver inflammation (Azarang et al., 2020). Probiotic mixtures containing *L. acidophilus* NCIMB 30175, *L. plantarum* NCIMB 30173, *L. rhamnosus* NCIMB 30174, and *Enterococcus faecium* NCIMB 30176 have also been shown to modulate intestinal immunity by inhibiting the release of pro-inflammatory cytokines such as IL-8, CXCL10, and CCL2 (Moens et al., 2019). *Lactobacillus paracasei*, a bacterium used in food processing and production, can persist in the human intestinal environment and combat obesity and metabolic disorders induced by a HFD (Tazi et al., 2018). This bacterium can also reduce NASH-associated inflammation by modulating liver M1/M2 states and strengthening intestinal barrier function, ultimately alleviating hepatic lipid accumulation and inflammation (Sohn et al., 2015). Furthermore, the *L. pentosus* strain S-PT84 interacts with intestinal Th17 cells to enhance intestinal tight junctions, alleviating the inflammation response, and directly modulates liver M1/M2 homeostasis in NAFLD mice, interfering with hepatic immunity and improving hepatic inflammation (Sakai et al., 2020).

*Lactobacillus plantarum*, prevalent in both soil and human intestines, holds potential as a probiotic, playing a role in hepatic macrophage polarization. *In vitro* studies have shown that *L. plantarum*-derived EVs can relieve skin inflammation by promoting differentiation of human monocytes into anti-inflammatory M2 macrophages (Kim et al., 2020). Diverse *L. plantarum* strains have been implicated in NAFLD development and progression. In HFD-induced obesity models, the *L. plantarum* N07 strain mitigates inflammation by significantly reducing the secretion of pro-inflammatory cytokines TNF-α and IL-1β, and by decreasing macrophage markers Cd11c and F4/80 in epididymal adipose tissue, thereby alleviating obesity-associated inflammatory injury (Yin et al., 2020). The *L. plantarum* NA136 strain, isolated from traditional pickles, modulates fatty acid metabolism and improves lipid accumulation in hepatocytes via the AMPK pathway, while also promoting the expression of antioxidant and detoxification enzymes through the NF-E2-related factor 2 (Nrf2) pathway to alleviate oxidative stress (Zhao et al., 2019). Similarly, the *L. plantarum* NCU116 strain, a newly identified probiotic isolated from pickled vegetables, mitigates liver steatosis and oxidative stress in HFD-induced NAFLD rodents by up-regulating genes related to fatty acid metabolism and decomposition (Li et al., 2014). Collectively, the findings suggest that *Lactobacillus* strains are integral to NAFLD treatment, both through hepatic lipid regulation and oxidative stress mitigation, and by directly balancing M1/M2 hepatic macrophages to regulate immunity and reduce inflammation.

### 4.4 Intestinal microbe-related metabolites adjust hepatic macrophage phenotypes

The ability of intestinal microbiota to regulate hepatic macrophage polarization is well established. Research indicates that metabolites associated with intestinal microbiota, including SCFAs, indole derivatives, LPS, and ethanol, can influence hepatic macrophage polarization.

#### 4.4.1 Intestinal microbe-related metabolites inhibit hepatic macrophage polarization to M1 phenotype

Butyrate, primarily derived from a high-fiber diet, serves as the principal energy source for the intestinal epithelium and acts as an anti-inflammatory agent. This compound can strengthen the intestinal barrier and reduce the translocation of gut-derived LPS to the liver (Al Bander et al., 2020). In both patients and mice with NAFLD, there is a marked reduction in hepatic GLP-1 and GLP-1R expression; however, exogenous butyrate supplementation not only reverses this trend but also modulates blood glucose metabolism and reduces hepatic steatosis and inflammation (Chen and Vitetta, 2020). Butyrate predominantly originates from *Faecalibacterium prausnitzii*, which exhibits a negative correlation with the KC marker CD163^+^. Similarly, in the portal vein of NAFLD patients, the expression levels of KCs and CD163^+^ are significantly elevated, suggesting a suppressive effect of butyrate on KCs (Schwenger et al., 2018; Wang Y. et al., 2021).

Indole, an intestinal microbial metabolite, exhibits prominent anti-inflammatory effects, alleviating hepatic steatosis and inflammation in mice with MCD-induced NAFLD (Zhu et al., 2022). Indole-3-acetic acid (IAA), a derivative of tryptophan metabolites produced by intestinal microbiota, is substantially reduced in HFD-fed mice. Krishnan et al. demonstrated that IAA interacts with the aryl hydrocarbon receptor (AhR) and modulates gene expression related to fatty acid metabolism, thereby ameliorating hepatic steatosis (Krishnan et al., 2018). Overwhelming evidence has demonstrated that the functions of palmitic acid and LPS are closely associated with hepatic macrophage activation, but their effects can be counteracted by IAA. Notably, IAA exerts significantly immunomodulatory effects by decreasing the production of pro-inflammatory cytokines, such as TNF-α and IL-1β, in macrophages pretreated with palmitic acid and LPS, ultimately inhibiting hepatic M1 macrophage activation *in vitro*. More importantly, IAA can directly target the CCL2-CCR2 signaling pathway and inhibit CCL2 secretion from the liver, thus suppressing MoMφs liver recruitment and improving hepatocyte lipid accumulation and hepatic inflammation (Krishnan et al., 2018). Bariatric surgery, effective for sustained weight loss and treatment of NAFLD in patients with severe obesity, results in elevated IAA levels in serum, stool, and liver biopsies, with post-surgical enterogenous-IAA translocation into the liver moderating M1/M2 states and ameliorating obesity-associated NAFLD (Wang Y. et al., 2021).

#### 4.4.2 Intestinal microbe-related metabolites contribute to hepatic macrophage polarization to M1 phenotype

Originating from the cell outer membrane of gram-negative bacteria, LPS serves as a classical stimulant for macrophage activation. Its interaction with the LR4/MyD88/NF-κB signaling pathway leads to elevated secretion of inflammatory cytokines and activation of M1 macrophages, thus promoting NAFLD development (Carpino et al., 2020). Various endotoxin-producing bacteria, such as *Enterobacter cloacae* B29, *E. coli* PY102, and *Klebsiella pneumoniae* A7, proliferate in the intestines of obese NAFLD patients (Chen and Vitetta, 2020). Ethanol, another potent pro-inflammatory stimulus, can promote the recruitment of pro-inflammatory MoMφs to the liver in rodents (Dou et al., 2019). Research has indicated that high-alcohol-producing *Klebsiella pneumoniae* is prevalent in the intestines of NAFLD patients and produces excessive endogenous alcohol. Experiments have also revealed that introducing high-alcohol-producing *Klebsiella pneumoniae* to 6-week-old C57B/6 J mice can increase the expression of the CYP2E1 protein and infiltration of M1 macrophages, neutrophils, and other immune cells in the liver, ultimately aggravating NAFLD progression (Yuan et al., 2019).

### 4.5 FMT remodulate hepatic macrophage phenotypes

Fecal microbiota transplantation (FMT) is an emerging and underexplored approach to restoring the gut microbiota to a balanced state (Ooijevaar et al., 2019). FMT offers a broader spectrum of intestinal symbiotic bacteria and has been extensively utilized in the treatment of chronic liver disease (Abenavoli et al., 2022). FMT significantly suppresses pro-inflammatory cytokines that associated with M1 macrophages, activates anti-inflammatory cytokines of IL-4 and IL-22 related to M2 macrophages meanwhile, thereby collectively improves hepatic histopathological manifestations in NAFLD mice (Zhou et al., 2017).

The safety and tolerability of FMT application on NAFLD are supported by several preclinical and clinical evidences (Vrieze et al., 2012; Witjes et al., 2020). Allogeneic FMT significantly rises an insulin sensitivity in 6 weeks, because butyrate-producing bacteria effectively prevent the endotoxic compounds translocation, that derived from the gut microbes (Vrieze et al., 2012). Witjes et al. (2020) also highlight the advantages of allogeneic FMT in NAFLD owed to the reduced proinflammation metabolites and lipid metabolism in plasma. Additionally, FMT effectively inhibits liver fat accumulation and repairs the gut dysbiosis in NAFLD patients (Xue et al., 2022). To summarize, FMT is a promising therapeutic approach for NASH by improving gut microbiota homeostasis, intestinal barrier function and inflammatory cytokine profiling. Collectively, this intervention holds valuable potential for ameliorating the pathogenesis and progression of NAFLD/NASH.

**Figure 3:**
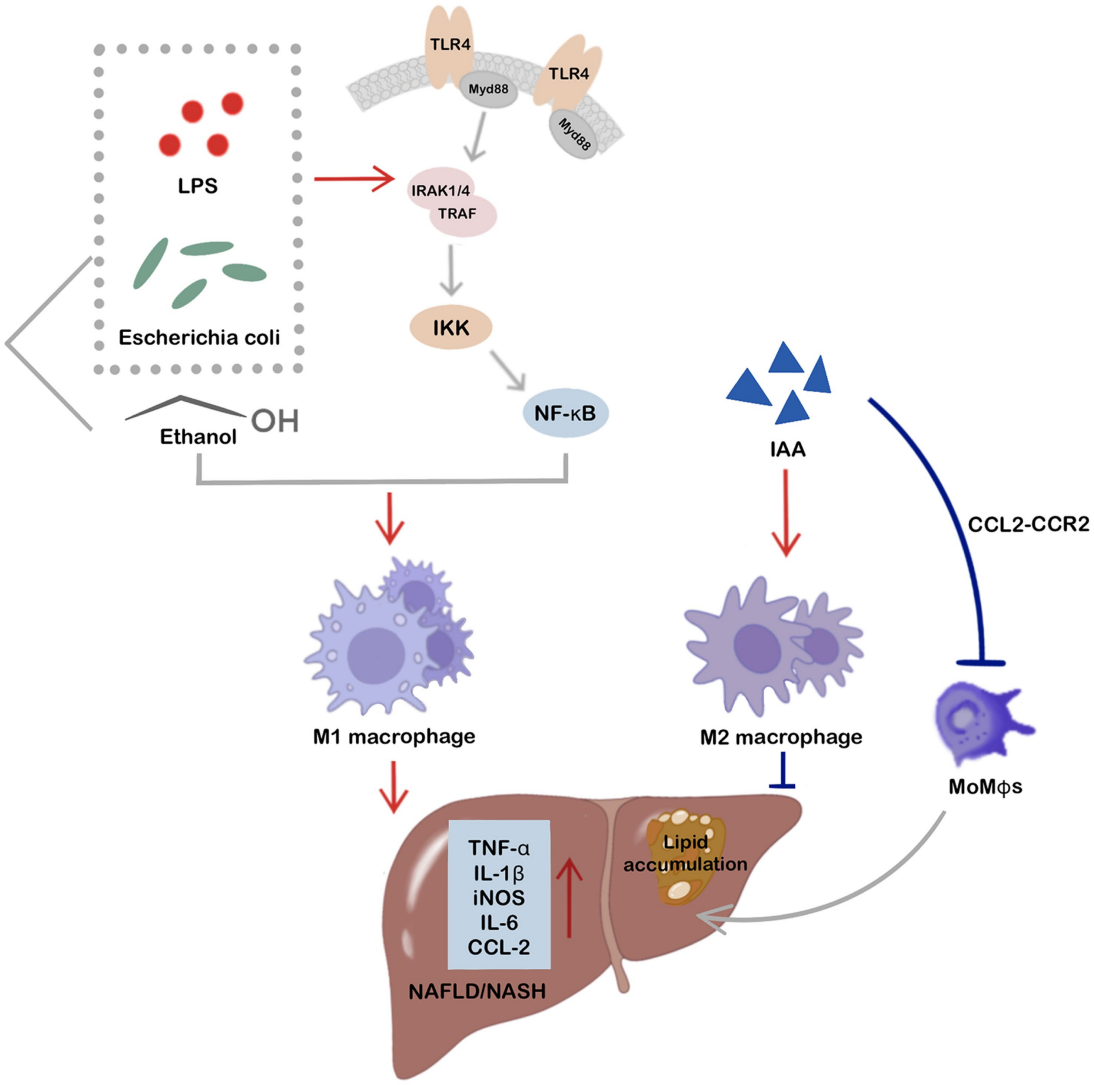
Intestinal microbes and related metabolites, including *Escherichia coli*, LPS, ethanol, and IAA, show direct involvement in the regulation of hepatic macrophage phenotypes and participate in NAFLD after translocation to the liver via the gut-liver axis. Notably, *E. coli*, LPS, and ethanol are potent stimuli for hepatic M1 macrophage activation, ultimately promoting inflammation and NAFLD/NASH progression. LPS and *E. coli* can activate hepatic M1 macrophages via the TLR4/MyD88/NF-κB signaling pathway. IAA, a tryptophan metabolite of intestinal microbes, not only activates hepatic M2 macrophages to improve hepatic inflammation and NAFLD, but also directly targets the CCL2-CCR2 signaling pathway, thereby inhibiting MoMφs recruitment to the liver and improving hepatocyte fat accumulation and hepatic inflammation.

In conclusion, these findings strongly suggest that gut-derived microbiota and their metabolites translocate to liver and crosstalk with hepatic macrophages, playing a crucial role in the development and progression of NAFLD/NASH. Targeting hepatic macrophage polarization by modulating intestinal microbes and their associated metabolites holds considerable potential for NAFLD treatment. While current research on the translocation of these intestinal components and their direct influence on hepatic macrophage phenotype remains limited, research has indicated that *E. coli*, LPS, ethanol, and IAA can directly regulate macrophage phenotypes after translocation into the liver via the gut-liver axis, thereby impacting NAFLD development and progression (Figure 3).

## 5 Conclusion

Hepatic macrophages, serving as the first line of defense against bacterial invasion, assume a key role in the development of NAFLD. They possess the capacity for phenotypic polarization, diverging into anti-inflammatory and pro-inflammatory states contingent upon the environmental and exogenous stimuli encountered. Disruption of the intestinal microbiota impacts hepatic immunity through the gut-liver axis, emerging as an important factor in NAFLD/NASH progression. Currently, approved pharmacotherapeutic interventions for NAFLD/NASH treatment are lacking. Although dietary modifications and exercise exhibit promise as remedies for NAFLD/NASH treatment, their efficacy diminishes in patients with morbid obesity and poor adherence.

NASH is a heterogeneous disease with multiple adverse factors contributing to its development. Activation of hepatic macrophages and dysbiosis of intestinal microbiota are typical features of NAFLD. When hepatic disease occurs, intestinal microbial dysbiosis disrupts the intestinal barrier, leading to the translocation of intestinal microbes and their metabolites to the liver via the gut-liver axis, which activates hepatic macrophages. Consequently, innovative therapeutics for NAFLD/NASH may necessitate interventions targeting hepatic macrophage phenotypic modulation via regulation of the intestinal microbiota. Several studies have revealed the direct regulatory impact of intestinally derived microorganisms and their metabolites, translocated through the gut-liver axis, upon hepatic macrophage phenotypes. However, investigations delving into the direct modulation of macrophage phenotypes by the intestinal microbiota remain insufficient. Relevant research has demonstrated that intestinal microbiota can adjust hepatic macrophage phenotypes and improve hepatic inflammation through exogenous supplementation. Nevertheless, conclusive evidence regarding the direct translocation of intestinal microbiota to the liver, orchestrating direct regulation of hepatic macrophage polarization, remains inadequate. Furthermore, studies elucidating the relevant signaling pathways remain insufficiently comprehensive.

Probiotics, prebiotics, and biogenics have garnered significant attention within NAFLD research due to their potential in mitigating NAFLD and NASH by fortifying the intestinal barrier and modulating hepatic immune responses. Nonetheless, the gut-liver axis encounters disruption in the presence of NAFLD. Upon exogenous administration of these agents, their entry into the liver via the gut-liver axis raises questions about potential exacerbation of hepatic injury. Thus, through comprehensive discussion of the established mechanisms involved in the direct activation of hepatic macrophages after translocation of gut-originating microorganisms and their metabolites to the liver during NAFLD onset, this review aims to offer insights for the future development of small-molecule targeted therapeutics.

## Author contributions

YC: Validation, Writing – original draft, Writing – review & editing. YG: Software, Validation, Writing – review & editing. HZ: Validation, Writing – review & editing. YL: Validation, Writing – review & editing. JH: Validation, Writing – review & editing. WW: Conceptualization, Funding acquisition, Supervision, Writing – original draft, Writing – review & editing. JG: Conceptualization, Funding acquisition, Validation, Writing – original draft, Writing – review & editing.

## Glossary

3IPA: 3-Indole propionic acid
*A. muciniphila*: *Akkermansia muciniphila*
AhR: Aryl hydrocarbon receptor
APCs: Antigen-presenting cells
Arg1: Arginase 1
CCL2: chemokine cytokine ligand 2
CCR2: chemokine cytokine receptor 2
Clec4e: C-type lectin domain family 4 member E
NAFLD: Non-alcoholic fatty liver disease
DCs: Dendritic cells
*E. coli*: *Escherichia coli*
*E. rectale*: *Eubacterium rectale*
Evs: Extracellular vesicles
FMT: Fecal microbiota transplantation
*F. prausnitzii*: *Faecalibacterium prausnitzii*
GLP-1: Glucagon-like peptide-1
GLP-1R: Glucagon-like peptide-1R
GVB: Gut-vascular barrier
HCC: Hepatocellular carcinoma
HFD: High-fat diet
HFHC: High-fat/high-cholesterol
IAA: Indole-3-acetic acid
IFN-γ: Interferon γ
IL-17: Interleukin 17
iNOS: Inducible nitric oxide synthase
KCs: Kupffer cells
LPS: Lipopolysaccharide
MHC-II: Major histocompatibility complex-II
MoMφs: Monocyte-derived macrophages
mtDNA: Mitochondrial DNA
MyD88: Myeloid differentiation factor 88
NAFL: Non-alcoholic fatty liver
NASH: Non-alcoholic steatohepatitis
NK cells: Natural killer cells
NLRP3: NOD-like receptor thermal protein domain associated protein 3
Nrf2: NF-E2-related factor 2
OS: Oxidative stress
PAMPs: Pathogen-associated molecular patterns
PRRs: Pattern recognition receptors
PV1: Plasmalemma vesicle-associated protein 1
Treg cells: Regulatory T cells
SCFAs: Short-chain fatty acids
SREBP-1c: Sterol regulatory element binding protein-1c
T2DM: Type 2 diabetes mellitus
TGF-β: Transform growth factor-β
Th17 cells: T helper 17 cells
TLR4: Toll-like receptor 4
TNF-α: Tumor necrosis factor-α

## References

[ref1] Abenavoli L. Maurizi V. Rinninella E. Tack J. Di Berardino A. Santori P.. (2022). Fecal microbiota transplantation in NAFLD treatment. Medicina 58:1559. doi: 10.3390/medicina58111559 36363516 PMC9695159

[ref2] Al Bander Z. Nitert M. D. Mousa A. Naderpoor N. (2020). The gut microbiota and inflammation: An overview. Int. J. Environ. Res. Public Health 17:7618. doi: 10.3390/ijerph17207618, PMID: 33086688 PMC7589951

[ref3] Albillos A. de Gottardi A. Rescigno M. (2020). The gut-liver axis in liver disease: pathophysiological basis for therapy. J. Hepatol. 72, 558–577. doi: 10.1016/j.jhep.2019.10.003 31622696

[ref4] Aron-Wisnewsky J. Warmbrunn M. V. Nieuwdorp M. Clément K. (2020). Nonalcoholic fatty liver disease: modulating gut microbiota to improve severity? Gastroenterology 158, 1881–1898. doi: 10.1053/j.gastro.2020.01.049 32044317

[ref5] Azarang A. Farshad O. Ommati M. M. Jamshidzadeh A. Heydari R. Abootalebi S. N.. (2020). Protective role of probiotic supplements in hepatic steatosis: a rat model study. Biomed. Res. Int. 2020, 1–15. doi: 10.1155/2020/5487659 PMC7704153 33299871

[ref6] Barrow F. Khan S. Fredrickson G. Wang H. Dietsche K. Parthiban P.. (2021). Microbiota-driven activation of intrahepatic B cells aggravates NASH through innate and adaptive signaling. Hepatology 74, 704–722. doi: 10.1002/hep.31755 33609303 PMC8377092

[ref7] Bruzzì S. Sutti S. Giudici G. Burlone M. E. Ramavath N. N. Toscani A.. (2018). B2-lymphocyte responses to oxidative stress-derived antigens contribute to the evolution of nonalcoholic fatty liver disease (NAFLD). Free Radic. Biol. Med. 124, 249–259. doi: 10.1016/j.freeradbiomed.2018.06.015, PMID: 29920340

[ref8] Cani P. D. Depommier C. Derrien M. Everard A. de Vos W. M. (2022). *Akkermansia muciniphila*: paradigm for next-generation beneficial microorganisms. Nat. Rev. Gastroenterol. Hepatol. 19, 625–637. doi: 10.1038/s41575-022-00631-9 35641786

[ref9] Carpino G. Del Ben M. Pastori D. Carnevale R. Baratta F. Overi D.. (2020). Increased liver localization of lipopolysaccharides in human and experimental NAFLD. Hepatology 72, 470–485. doi: 10.1002/hep.31056 31808577

[ref10] Chassaing B. Compher C. Bonhomme B. Liu Q. Tian Y. Walters W.. (2022). Randomized controlled-feeding study of dietary emulsifier Carboxymethylcellulose reveals detrimental impacts on the gut microbiota and metabolome. Gastroenterology 162, 743–756. doi: 10.1053/j.gastro.2021.11.006 34774538 PMC9639366

[ref11] Chen W. Liu Y. Chen J. Ma Y. Song Y. Cen Y.. (2021). The notch signaling pathway regulates macrophage polarization in liver diseases. Int. Immunopharmacol. 99:107938. doi: 10.1016/j.intimp.2021.107938 34371331

[ref12] Chen J. Vitetta L. (2020). Gut microbiota metabolites in NAFLD pathogenesis and therapeutic implications. Int. J. Mol. Sci. 21:5214. doi: 10.3390/ijms21155214 32717871 PMC7432372

[ref13] Depommier C. Everard A. Druart C. Plovier H. Van Hul M. Vieira-Silva S.. (2019). Supplementation with *Akkermansia muciniphila* in overweight and obese human volunteers: a proof-of-concept exploratory study. Nat. Med. 25, 1096–1103. doi: 10.1038/s41591-019-0495-2, PMID: 31263284 PMC6699990

[ref14] Dou L. Shi X. He X. Gao Y. (2019). Macrophage phenotype and function in liver disorder. Front. Immunol. 10:3112. doi: 10.3389/fimmu.2019.03112 32047496 PMC6997484

[ref15] Govaere O. Petersen S. K. Martinez-Lopez N. Wouters J. Van Haele M. Mancina R. M.. (2022). Macrophage scavenger receptor 1 mediates lipid-induced inflammation in non-alcoholic fatty liver disease. J. Hepatol. 76, 1001–1012. doi: 10.1016/j.jhep.2021.12.012, PMID: 34942286 PMC7619241

[ref16] Han Y. Li L. Wang B. (2022). Role of *Akkermansia muciniphila* in the development of nonalcoholic fatty liver disease: current knowledge and perspectives. Front. Med. 16, 667–685. doi: 10.1007/s11684-022-0960-z 36318353

[ref17] Kazankov K. Jorgensen S. M. D. Thomsen K. L. Moller H. J. Vilstrup H. George J.. (2019). The role of macrophages in nonalcoholic fatty liver disease and nonalcoholic steatohepatitis. Nat. Rev. Gastroenterol. Hepatol. 16, 145–159. doi: 10.1038/s41575-018-0082-x 30482910

[ref18] Keshavarz Azizi Raftar S. Ashrafian F. Yadegar A. Lari A. Moradi H. R. Shahriary A.. (2021). The protective effects of live and pasteurized Akkermansia muciniphila and its extracellular vesicles against HFD/CCl4-induced liver injury. Microbiol Spectr 9:e0048421. doi: 10.1128/Spectrum.00484-21, PMID: 34549998 PMC8557882

[ref19] Kim W. Lee E. J. Bae I. H. Myoung K. Kim S. T. Park P. J.. (2020). *Lactobacillus plantarum*-derived extracellular vesicles induce anti-inflammatory M2 macrophage polarization in vitro. J. Extracell Vesicles 9:1793514. doi: 10.1080/20013078.2020.1793514 32944181 PMC7480564

[ref20] Krenkel O. Puengel T. Govaere O. Abdallah A. T. Mossanen J. C. Kohlhepp M.. (2018). Therapeutic inhibition of inflammatory monocyte recruitment reduces steatohepatitis and liver fibrosis. Hepatology 67, 1270–1283. doi: 10.1002/hep.29544, PMID: 28940700

[ref21] Krishnan S. Ding Y. Saedi N. Choi M. Sridharan G. V. Sherr D. H.. (2018). Gut microbiota-derived tryptophan metabolites modulate inflammatory response in hepatocytes and macrophages. Cell Rep. 23, 1099–1111. doi: 10.1016/j.celrep.2018.03.109 29694888 PMC6392449

[ref22] Li C. Nie S. P. Zhu K. X. Ding Q. Li C. Xiong T.. (2014). *Lactobacillus plantarum* NCU116 improves liver function, oxidative stress and lipid metabolism in rats with high fat diet induced non-alcoholic fatty liver disease. Food Funct. 5, 3216–3223. doi: 10.1039/C4FO00549J 25317840

[ref23] Liu N. Chang C. W. Steer C. J. Wang X. W. Song G. (2022). MicroRNA-15a/16-1 prevents hepatocellular carcinoma by disrupting the communication between Kupffer cells and regulatory T cells. Gastroenterology 162, 575–589. doi: 10.1053/j.gastro.2021.10.015 34678217

[ref24] Moens F. Van den Abbeele P. Basit A. W. Dodoo C. Chatterjee R. Smith B.. (2019). A four-strain probiotic exerts positive immunomodulatory effects by enhancing colonic butyrate production in vitro. Int. J. Pharm. 555, 1–10. doi: 10.1016/j.ijpharm.2018.11.020 30445175

[ref25] Mouries J. Brescia P. Silvestri A. Spadoni I. Sorribas M. Wiest R.. (2019). Microbiota-driven gut vascular barrier disruption is a prerequisite for non-alcoholic steatohepatitis development. J. Hepatol. 71, 1216–1228. doi: 10.1016/j.jhep.2019.08.005, PMID: 31419514 PMC6880766

[ref26] Nagashimada M. Honda M. (2021). Effect of microbiome on non-alcoholic fatty liver disease and the role of probiotics, prebiotics, and biogenics. Int. J. Mol. Sci. 22:8008. doi: 10.3390/ijms22158008 34360773 PMC8348401

[ref27] Natsui K. Tsuchiya A. Imamiya R. Osada-Oka M. Ishii Y. Koseki Y.. (2023). *Escherichia coli*-derived outer-membrane vesicles induce immune activation and progression of cirrhosis in mice and humans. Liver Int. 43, 1126–1140. doi: 10.1111/liv.15539 36751961

[ref28] Ooijevaar R. E. Terveer E. M. Verspaget H. W. Kuijper E. J. Keller J. J. (2019). Clinical application and potential of fecal microbiota transplantation. Annu. Rev. Med. 70, 335–351. doi: 10.1146/annurev-med-111717-122956 30403550

[ref29] Pan J. Ou Z. Cai C. Li P. Gong J. Ruan X. Z.. (2018). Fatty acid activates NLRP3 inflammasomes in mouse Kupffer cells through mitochondrial DNA release. Cell. Immunol. 332, 111–120. doi: 10.1016/j.cellimm.2018.08.006, PMID: 30103942

[ref30] Potoupni V. Georgiadou M. Chatzigriva E. Polychronidou G. Markou E. Zapantis Gakis C.. (2021). Circulating tumor necrosis factor-alpha levels in non-alcoholic fatty liver disease: a systematic review and a meta-analysis. J. Gastroenterol. Hepatol. 36, 3002–3014. doi: 10.1111/jgh.15631 34289181

[ref31] Riaz F. Wei P. Pan F. (2022). Fine-tuning of regulatory T cells is indispensable for the metabolic steatosis-related hepatocellular carcinoma: a review. Front. Cell Dev. Biol. 10:949603. doi: 10.3389/fcell.2022.949603 35912096 PMC9337771

[ref32] Sakai Y. Arie H. Ni Y. Zhuge F. Xu L. Chen G.. (2020). *Lactobacillus pentosus* strain S-PT84 improves steatohepatitis by maintaining gut permeability. J. Endocrinol. 247, 169–181. doi: 10.1530/JOE-20-0105, PMID: 33032263

[ref33] Schirmer M. Smeekens S. P. Vlamakis H. Jaeger M. Oosting M. Franzosa E. A.. (2016). linking the human gut microbiome to inflammatory cytokine production capacity. Cells 167, 1125–1136.e8. doi: 10.1016/j.cell.2016.10.020 PMC5131922 27814509

[ref34] Schwenger K. J. P. Chen L. Chelliah A. Da Silva H. E. Teterina A. Comelli E. M.. (2018). Markers of activated inflammatory cells are associated with disease severity and intestinal microbiota in adults with non-alcoholic fatty liver disease. Int. J. Mol. Med. 42, 2229–2237. doi: 10.3892/ijmm.2018.3800 30085339

[ref35] Slevin E. Baiocchi L. Wu N. Ekser B. Sato K. Lin E.. (2020). Kupffer cells: inflammation pathways and cell-cell interactions in alcohol-associated liver disease. Am. J. Pathol. 190, 2185–2193. doi: 10.1016/j.ajpath.2020.08.014 32919978 PMC7587925

[ref36] Sohn W. Jun D. W. Lee K. N. Lee H. L. Lee O. Y. Choi H. S.. (2015). *Lactobacillus paracasei* induces M2-dominant Kupffer cell polarization in a mouse model of nonalcoholic steatohepatitis. Dig. Dis. Sci. 60, 3340–3350. doi: 10.1007/s10620-015-3770-1 26143342

[ref37] Somm E. Montandon S. A. Loizides-Mangold U. Gaia N. Lazarevic V. De Vito C.. (2021). The GLP-1R agonist liraglutide limits hepatic lipotoxicity and inflammatory response in mice fed a methionine-choline deficient diet. Transl. Res. 227, 75–88. doi: 10.1016/j.trsl.2020.07.008 32711187

[ref38] Tazi A. Araujo J. R. Mulet C. Arena E. T. Nigro G. Pedron T.. (2018). Disentangling host-microbiota regulation of lipid secretion by enterocytes: insights from commensals Lactobacillus paracasei and *Escherichia coli*. MBio 9:e01493-18. doi: 10.1128/mBio.01493-18 30181250 PMC6123438

[ref39] Vrieze A. Van Nood E. Holleman F. Salojärvi J. Kootte R. S. Bartelsman J. F.. (2012). Transfer of intestinal microbiota from lean donors increases insulin sensitivity in individuals with metabolic syndrome. Gastroenterology 143, 913–6.e7. doi: 10.1053/j.gastro.2012.06.031 22728514

[ref40] Wang C. Ma C. Gong L. Guo Y. Fu K. Zhang Y.. (2021). Macrophage polarization and its role in liver disease. Front. Immunol. 12:803037. doi: 10.3389/fimmu.2021.803037, PMID: 34970275 PMC8712501

[ref41] Wang Y. Wang G. Bai J. Zhao N. Wang Q. Zhou R.. (2021). Role of Indole-3-acetic acid in NAFLD amelioration after sleeve gastrectomy. Obes. Surg. 31, 3040–3052. doi: 10.1007/s11695-021-05321-0 33973136

[ref42] Wen Y. Lambrecht J. Ju C. Tacke F. (2021). Hepatic macrophages in liver homeostasis and diseases-diversity, plasticity and therapeutic opportunities. Cell. Mol. Immunol. 18, 45–56. doi: 10.1038/s41423-020-00558-8, PMID: 33041338 PMC7852525

[ref43] Witjes J. J. Smits L. P. Pekmez C. T. Prodan A. Meijnikman A. S. Troelstra M. A.. (2020). Donor fecal microbiota transplantation alters gut microbiota and metabolites in obese individuals with steatohepatitis. Hepatol. Commun. 4, 1578–1590. doi: 10.1002/hep4.1601 33163830 PMC7603524

[ref44] Wu H. M. Ni X. X. Xu Q. Y. Wang Q. Li X. Y. Hua J. (2020). Regulation of lipid-induced macrophage polarization through modulating peroxisome proliferator-activated receptor-gamma activity affects hepatic lipid metabolism via a toll-like receptor 4/NF-kappaB signaling pathway. J. Gastroenterol. Hepatol. 35, 1998–2008. doi: 10.1111/jgh.15025 32128893

[ref45] Xue L. Deng Z. Luo W. He X. Chen Y. (2022). Effect of fecal microbiota transplantation on non-alcoholic fatty liver disease: a randomized clinical trial. Front. Cell. Infect. Microbiol. 12:759306. doi: 10.3389/fcimb.2022.759306, PMID: 35860380 PMC9289257

[ref46] Yao Q. Li S. Li X. Wang F. Tu C. (2020). Myricetin modulates macrophage polarization and mitigates liver inflammation and fibrosis in a murine model of nonalcoholic steatohepatitis. Front. Med. 7:71. doi: 10.3389/fmed.2020.00071 PMC7065264 32195263

[ref47] Yin T. Bayanjargal S. Fang B. Inaba C. Mutoh M. Kawahara T.. (2020). *Lactobacillus plantarum* Shinshu N-07 isolated from fermented *Brassica rapa* L. attenuates visceral fat accumulation induced by high-fat diet in mice. Benef Microbes 11, 655–667. doi: 10.3920/BM2020.0009 33045842

[ref48] Yuan J. Chen C. Cui J. Lu J. Yan C. Wei X.. (2019). Fatty liver disease caused by high-alcohol-producing *Klebsiella pneumoniae*. Cell Metab. 30, 675–688.e7. doi: 10.1016/j.cmet.2019.08.018, PMID: 31543403

[ref49] Zhang X. Coker O. O. Chu E. S. Fu K. Lau H. C. H. Wang Y. X.. (2021). Dietary cholesterol drives fatty liver-associated liver cancer by modulating gut microbiota and metabolites. Gut 70, 761–774. doi: 10.1136/gutjnl-2019-319664 32694178 PMC7948195

[ref50] Zhang S. Gang X. Yang S. Cui M. Sun L. Li Z.. (2021). The alterations in and the role of the Th17/Treg balance in metabolic diseases. Front. Immunol. 12:678355. doi: 10.3389/fimmu.2021.678355 34322117 PMC8311559

[ref51] Zhang Y. Jiang W. Xu J. Wu N. Wang Y. Lin T.. (2020). E. coli NF73-1 isolated from NASH patients aggravates NAFLD in mice by translocating into the liver and stimulating M1 polarization. Front. Cell. Infect. Microbiol. 10:535940. doi: 10.3389/fcimb.2020.535940, PMID: 33363046 PMC7759485

[ref52] Zhao Z. Wang C. Zhang L. Zhao Y. Duan C. Zhang X.. (2019). *Lactobacillus plantarum* NA136 improves the non-alcoholic fatty liver disease by modulating the AMPK/Nrf2 pathway. Appl. Microbiol. Biotechnol. 103, 5843–5850. doi: 10.1007/s00253-019-09703-4 31115630

[ref53] Zhou D. Pan Q. Shen F. Cao H. X. Ding W. J. Chen Y. W.. (2017). Total fecal microbiota transplantation alleviates high-fat diet-induced steatohepatitis in mice via beneficial regulation of gut microbiota. Sci. Rep. 7:1529. doi: 10.1038/s41598-017-01751-y, PMID: 28484247 PMC5431549

[ref54] Zhu L. Baker S. S. Gill C. Liu W. Alkhouri R. Baker R. D.. (2013). Characterization of gut microbiomes in nonalcoholic steatohepatitis (NASH) patients: a connection between endogenous alcohol and NASH. Hepatology 57, 601–609. doi: 10.1002/hep.26093 23055155

[ref55] Zhu B. Li H. Lu B. Guo X. Wu C. Wang F.. (2022). Indole supplementation ameliorates MCD-induced NASH in mice. J. Nutr. Biochem. 107:109041. doi: 10.1016/j.jnutbio.2022.109041 35568098

